# Efficacy of High-Dose Methylprednisolone in Inducing Remission in Pediatric Acute Severe Ulcerative Colitis: Retrospective Study

**DOI:** 10.3390/jcm14144938

**Published:** 2025-07-12

**Authors:** Dominika Marszk, Aleksandra Treder, Agnieszka Szlagatys-Sidorkiewicz, Michał Brzeziński

**Affiliations:** 1 Department of Pediatrics, Copernicus Hospital, 80-803 Gdańsk, Poland; dsierdzinska@copernicus.gda.pl (D.M.); olagru1994@gmail.com (A.T.); 2 Department of Pediatrics, Gastroenterology, Allergology and Nutrition, Faculty of Medicine, Medical University of Gdańsk, 80-803 Gdańsk, Poland; agnieszka.szlagatys-sidorkiewicz@gumed.edu.pl

**Keywords:** pediatric inflammatory bowel disease, ulcerative colitis, steroid pulse therapy

## Abstract

**Background**: Ulcerative colitis (UC) is increasing in incidence, including among pediatric populations. Treatment aims primarily to induce and maintain remission. For those inadequately responding to 5-aminosalicylic acid, remission may be induced via oral steroids or, in severe instances, intravenous methylprednisolone. This retrospective case series aims to evaluate the efficacy of high-dose intravenous methylprednisolone pulses (30 mg/kg, max 1 g per day for 3–5 days) in inducing remission in moderate and severe pediatric UC cases. **Methods**: From a cohort of pediatric patients (<18 years) hospitalized in 2018–2021 due to an acute flare of UC, those treated with high doses of methylprednisolone to induce remission were identified. The Pediatric Ulcerative Colitis Activity Index (PUCAI) was used to determine the response to treatment, considering a 20-point reduction or a score below 10 as significant improvement and indicative of remission induction. **Results**: Disease activity was severe in most patients (12/15), with 3/15 having moderate but refractory disease. We observed a clinically significant response in 9/15 patients (60%) with a mean PUCAI decrease of 39.4 ± 14.7 points. The median duration to clinical remission was 4 (IQR 3–4) days. For the 6/15 non-responders to methylprednisolone pulses, treatment was escalated. Adverse effects were not observed during the treatment period. **Conclusions**: High-dose methylprednisolone may be a viable alternative for inducing remission in pediatric UC. However, the small sample size and retrospective design warrant further prospective studies to validate these findings.

## 1. Introduction

Ulcerative colitis (UC) is a chronic inflammatory bowel disease affecting the mucosa of the colon, extending proximally from the rectum. It most commonly presents with diarrhea, hematochezia, and abdominal pain [1]. The etiology of the disease is thought to involve multiple factors, including genetic, environmental, and immunological aspects. There is an increasing incidence of UC in both adults and children, with 20% of patients being under 20 years of age at the time of diagnosis [2]. In most European countries and the USA, the incidence of new cases among children is estimated to be 1–4 per 100,000 per year [3]. At the time of diagnosis, approximately 51% of patients with UC in the pediatric population present with extensive disease (E3 on the Montreal scale [4]), nearly twice the rate seen in the adult population [5]. Treatment aims to induce remission, followed by the maintenance of that remission and a reduction in the risk of disease complications and treatment-related issues.

According to ESPGHAN guidelines, oral glucocorticoid therapy is considered second-line treatment in patients with mild to moderate disease who do not respond to 5-aminosalicylic acid (5-ASA) therapy [6]. Due to the extent of lesions and the more severe disease course, systemic glucocorticoids (GCs) are often used in children and adolescents with UC to induce remission [7].

This approach is the standard in severe cases of the disease, as the efficacy of GCs was demonstrated in a randomized trial conducted in 1955 by Truelove and Witts in adult patients with UC [8].

The prolonged systemic administration of GCs is associated with numerous adverse effects. In children, particular attention is given to the impact of GCs on growth disorders, osteopenia, and Cushing’s syndrome [9].

Pediatric patients with UC have a significantly higher rate of steroid dependence compared to adult patients (45% vs. 8%, respectively) [10]. The failure of systemic steroid therapy necessitates the implementation of subsequent steps in the management of acute severe ulcerative colitis (ASUC). Current ESPGHAN guidelines recommend the use of biological and immunosuppressive therapies, as well as the consideration of surgical treatment options [11].

Infliximab demonstrates efficacy in inducing and maintaining remission in ASUC patients [12]. However, the response to TNF-alpha inhibitors may not occur until 1–4 weeks, peaking at 12–16 weeks [6]. The extended duration of action and altered pharmacokinetics in ASUC [12] contribute to a higher primary non-response rate compared to moderate disease [13].

Cyclosporine has 81% short-term efficacy in pediatric ASUC but 39% long-term success, warranting its use only as a bridge to thiopurines, while limited data on tacrolimus suggests 79% short-term but only 9% long-term efficacy [11].

Given the toxicity profile of cyclosporine and the limited availability of biological therapies, high-dose intravenous steroid pulse therapy has been utilized in our clinic in exceptional situations, with the consent of the patients’ legal guardians, when there was a lack of response to standard doses or severe presentation of their disease, following the example of other specialties [14]. This treatment has not previously been applied in gastroenterology nor supported by strong evidence [15,16,17,18]; however, its effectiveness has been demonstrated in nephrology [19,20,21], rheumatology [22], and neurology [23].

The aim of this study was to assess the efficacy of steroid pulse doses as a rescue therapy in cases where standard doses of steroids were ineffective in pediatric patients with exacerbations of UC.

## 2. Materials and Methods

A retrospective analysis was conducted on pediatric patients (<18 years of age) diagnosed with UC who were hospitalized at the Department of Pediatrics, Gastroenterology, Allergy, and Pediatric Nutrition at the Medical University of Gdańsk from 2018 to 2021. Inclusion criteria were as follows: patients under 18 years of age diagnosed with ulcerative colitis based on clinical, endoscopic, and histological criteria, who received high-dose methylprednisolone pulse therapy for an acute exacerbation between 2018 and 2021. Patients with severe disease activity and those with moderate activity refractory to standard therapy were included. Exacerbations were defined as a relapse or worsening of clinical symptoms (including increased stool frequency, bloody stools, abdominal pain, or significant deterioration in laboratory or endoscopic findings) necessitating the escalation or adjustment of ongoing medication [24]. All included patients received intravenous methylprednisolone pulse therapy at a dose of 30 mg/kg body weight (maximum 1 g/day) for 3–5 days to induce remission. High-dose methylprednisolone pulse therapy was initiated immediately on admission in patients presenting with severe disease activity or after 3–5 days of standard intravenous steroid treatment in cases with inadequate clinical response. There were no explicit exclusion criteria beyond an age over 18 years and cases in which pulse therapy was administered for diagnoses other than ulcerative colitis. Disease activity was evaluated using the Pediatric Ulcerative Colitis Activity Index (PUCAI) [25] before the initiation of treatment and after the completion of therapy. A clinically significant improvement is defined as a reduction of at least 20 points in the PUCAI score or an absolute score below 10 points [26].

Patients who achieved a decrease of 20 points on the PUCAI scale were classified into the responder group.

The primary outcome was the induction of clinical remission, defined as a reduction of at least 20 points in the PUCAI score or an absolute PUCAI score below 10 after therapy. The secondary outcomes included time to clinical remission, the rate of treatment escalation to second-line therapies, colectomy within one year of follow-up, and the occurrence of adverse events during pulse therapy.

Patients were continuously monitored during hospitalization. Serious adverse events (SAEs) were defined as life-threatening complications, ICU transfer, or the discontinuation of therapy due to toxicity. No SAEs occurred

Patients were followed for up to one year after receiving high-dose methylprednisolone therapy. Primary outcome assessment (clinical remission) was performed at the completion of pulse therapy (median 4 days). Secondary outcomes—including escalation to second-line therapy and colectomy—were recorded over the one-year follow-up period.

The normality of the sample distribution was assessed using the Shapiro–Wilk test. The *t*-test for dependent samples was applied if the data had a normal distribution. If the data did not have a normal distribution, the Wilcoxon signed-rank test would have been used instead of Student’s *t*-test. Significance was established at *p* < 0.05.

The obtained data were aggregated in Microsoft Excel^®^ (Microsoft, Redmond, WA, USA). Data were calculated and summarized using descriptive statistics. Statistical analysis was performed using RStudio v 3.6.2 (RStudio, PBC, Boston, MA, USA).

Ethical approval was waived by the Bioethics Committee for Scientific Research at the Medical University of Gdańsk (KB/557/2024-2025; 30 January 2025) in view of the retrospective nature of this study and because all the procedures being performed were part of routine care.

## 3. Results

From a cohort of 121 pediatric patients diagnosed with UC hospitalized at the clinic between 2018 and 2021, 15 patients with exacerbations of the underlying disease were identified, who received methylprednisolone pulse therapy.

Table 1 presents the clinical characteristics of the patients who received treatment. The mean age was 12 years ± 3 years and 6 months. Eight (53%) patients were female and seven (47%) male. Assessing the PUCAI score prior to treatment, 3 out of 15 (20%) patients exhibited moderate disease activity (35–64 points), while the remaining 12 (80%) had severe disease (≥65 points on the PUCAI scale).

The median duration of disease from diagnosis until administering the methylprednisolone pulse therapy was 4 months (IQR 1–7). In three patients, this treatment was initiated at the time of diagnosis; in six patients, it was initiated during the first exacerbation of the disease; and the remaining six experienced two or more exacerbations. Seven (47%) patients initially received standard-dose steroid therapy; however, due to a lack of response, high-dose methylprednisolone pulse therapy was administered. Eight (53%) patients immediately qualified for pulse therapy due to the severe presentation of the disease.

In the group of 15 patients, the PUCAI score before the methylprednisolone pulse therapy was 71.3 ± 15.9. A mean reduction in the PUCAI score of 26.0 ± 20.7 points was observed on the fourth day of treatment. The difference in PUCAI scores before and after the intervention followed a normal distribution. The value of Student’s *t*-test for dependent samples was t = 4.86; *p* = 0.00025, indicating that the difference in PUCAI scores before and after the therapy was statistically significant.

**Figure 1:**
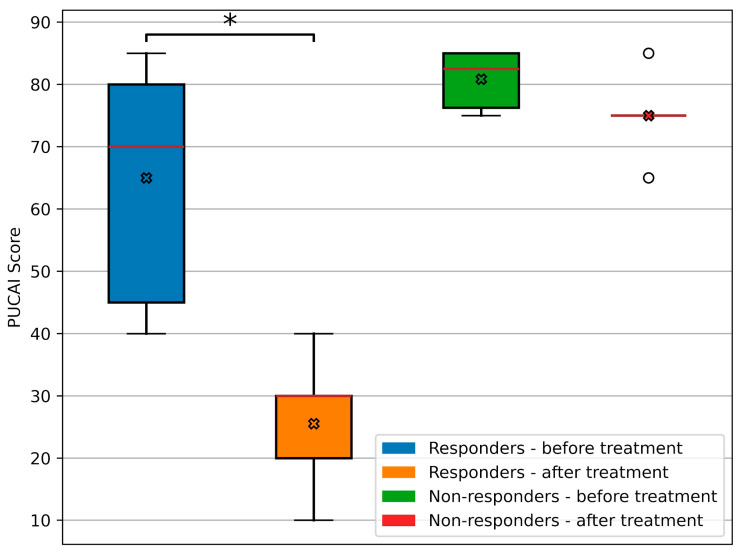
The distribution of PUCAI scores before and after treatment, along with the difference in scores before and after the intervention. * Indicates statistically significant differences (*p* < 0.05). x Indicates the mean. ◦ Indicates outliers.

In the analyzed cohort, a clinically and statistically significant response to treatment was observed in 9 out of 15 patients (60%) after the therapy was administered. Following the therapy, the change in the PUCAI score was statistically significant in the responder group but not in the non-responder group. On the fourth day of treatment, the PUCAI score for responders was 25.6 ± 9.2 points, while for non-responders, it was 75.0 ± 6.3 points. In the responder group, the mean decrease was 39.4 ± 14.7 points (*p*-value = 0.000041), indicating a statistically significant improvement. In the non-responder group, no statistically significant decrease was observed with a mean of 5.8 points (*p*-value = 0.0842). The changes in the PUCAI score are presented in Figure 1.

**Figure 2:**
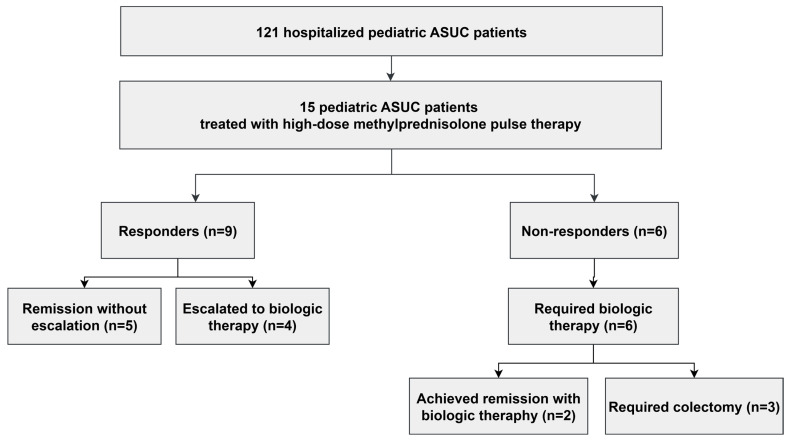
Flow diagram of treatment pathways and outcomes in 15 pediatric ASUC patients treated with high-dose methylprednisolone pulse therapy (2018–2021). Follow-up: 12 months. Counts indicate number of patients at each step. Abbreviation: ASUC, acute severe ulcerative colitis.

Patient observation following treatment lasted up to one year. Four out of nine responders, despite a good response to high doses of methylprednisolone, required the initiation of second-line therapy within 12 months of observation. Three out of nine patients received infliximab therapy, while one received vedolizumab. Five patients did not require the initiation of subsequent treatment during the 12-month observation period following steroid pulse therapy (Figure 2).

In the studied group, six (40%) patients did not respond to high-dose methylprednisolone therapy. Among these, five (33%) patients received biological therapy with infliximab, including one patient who had previously undergone cyclosporine treatment without improvement. Three (20%) patients did not achieve improvement after receiving rescue therapy with infliximab and required colectomy. One (7%) patient received adalimumab, resulting in an improvement in clinical status.

In the analyzed group of patients undergoing steroid pulse therapy, the careful observation and monitoring of vital signs were conducted, and no serious adverse effects of the treatment were reported.

## 4. Discussion

Current treatment guidelines for pediatric UC in moderate cases that do not respond to prior oral steroid therapy and in severe cases recommend the use of intravenous methylprednisolone at a dose of 1.5 mg/kg/day (maximum 60 mg/day) administered in one or two divided doses. If there is no response to intravenous corticosteroid (IVCS) treatment, infliximab (IFX) or calcineurin inhibitors (cyclosporine or tacrolimus) should be considered. Both options have been shown to be equally effective in inducing remission in ASUC; however, IFX is more commonly used due to better familiarity with the drug and the ability to continue IFX as maintenance therapy [11].

Currently, several different classes of advanced therapies have been approved for the treatment of inflammatory bowel diseases (IBDs): TNFα antagonists, anti-integrin medications, interleukin (IL)-12/23p40 antagonists, oral small molecule Janus kinase inhibitors, sphingosine-1-phosphate receptor modulators, and biological agents such as selective interleukin-23 antagonists [26]. Unfortunately, despite favorable study results in adult patient populations, most of these therapies have not yet been authorized for use in children with IBD.

In cases of the ineffectiveness of second-line treatment for ASUC or the development of complications such as acute colonic distension, perforation, or gastrointestinal bleeding, emergency colectomy may be necessary. Subsequently, restorative proctocolectomy with ileal pouch–anal anastomosis can be performed as a second stage of treatment. Emergency procedures carry a higher risk of early and late complications compared to elective colectomy [27].

Another clinically significant aspect of inducing remission is the time from the initiation of therapy to the reduction in disease symptoms. In children, the prediction of treatment failure with IVCSs is based on the PUCAI scale scores on days 3 and 5 of therapy. A PUCAI score above 45 points on day 3 should prompt the planning of second-line therapy introduction on day 5; a PUCAI score >65 points, and certainly >70 points, should lead to the execution of planned therapy. Current guidelines recommend initiating second-line therapy in cases of no response during 3–10 days of IVCS treatment [28]. In our study, a response to treatment was achieved in a median of 4 (IQR 3–4) days, allowing for the prompt initiation of the next treatment phase.

There are few reports in the literature regarding the advantages of using higher doses of intravenous corticosteroids compared to the standard doses recommended by ESPGHAN. In the study by Kudo et al. [15] conducted in Japan among 37 children with UC, the effectiveness of the initiating therapy was demonstrated in all patients receiving glucocorticoid therapy; however, patients receiving methylprednisolone in steroid pulses achieved remission more quickly compared to those receiving the drug in standard doses of 1–1.5 mg/kg/day (13.2 days vs. 25.1 days; *p* < 0.05). The dosing regimen differed from that used in our population, which involved a 3-day pulse therapy followed by the continuation of prednisolone for 4 days, with the sequence repeated up to three times until remission was achieved. In the study by Vora et al. [17], lower PUCAI scores were observed on the fifth day of treatment with IVCSs in the group receiving high-dose glucocorticoids compared to the group receiving lower doses (15 (IQR 7.5–17.5) vs. 25 (IQR 17.5–27.5)). The group using steroid pulses also reported a lower rate of colectomy. Conversely, in the study by Rosenberg et al. [16], comparing a group of 20 patients undergoing steroid pulse therapy with historical data from patients treated with standard doses of glucocorticoids, no advantage of methylprednisolone pulse therapy over standard dosing was demonstrated. In the study by Oshitani et al. [18], improvement in disease symptoms was noted within 7 days of high-dose intravenous methylprednisolone therapy (500 mg/day) compared to a much later onset of improvement—14 days—when using conventional therapy (0.8 mg oral prednisolone/kg body weight). However, no statistically significant difference between the groups was observed due to the small number of patients included.

The immunosuppressive, anti-inflammatory, and anti-allergic effects of glucocorticoids are mediated through genomic and non-genomic mechanisms [14]. The genomic mechanism involves receptor interaction and gene expression, while high doses of glucocorticoids can rapidly exert effects through physicochemical interactions with cell membranes. High-dose corticosteroids downregulate immune cell activation, reduce proinflammatory cytokines, and decrease neutrophil migration, similar to anti-TNF-alpha antibodies. While glucocorticoids have adverse effects, high-dose intravenous methylprednisolone aims to achieve faster and more potent efficacy with lower toxicity compared to oral prednisone, demonstrating effectiveness and safety in various conditions [29].

While there is a strong trend toward reducing corticosteroid use in pediatric IBD due to known adverse effects, high-dose pulse therapy may represent a strategy to limit cumulative steroid exposure in selected severe cases by achieving faster disease control and avoiding prolonged tapering regimens. This approach must be weighed against the risk of adverse events, and further studies are needed to confirm its safety and efficacy. In low-resource settings where access to biologics is limited, it may offer a pragmatic alternative to control disease activity and delay surgery.

Another aspect that requires further investigation is the comparison of other remission-inducing medications relative to high-dose intravenous glucocorticoids.

Cyclosporine (CyA) has demonstrated short-term efficacy as a rescue therapy in pediatric ASUC, with an 81% success rate (i.e., absence of colectomy) in the short term. However, its long-term success is limited, as only 39% of patients avoided surgical intervention [11,30]. Additionally, cyclosporine has a high toxicity profile, with serious adverse effects reported in both adult and pediatric populations, including nephrotoxicity, infections, and neurological complications.

Tacrolimus has emerged as a treatment option for UC, particularly in patients with steroid-resistant and steroid-dependent forms of the disease. Some trials conducted in adult populations indicated a significantly higher clinical response after 2 weeks of treatment compared to placebo with high colectomy-free rates in the following months [31]. In studies involving pediatric populations, short-term response rates ranged from 60% to 90%; however at least 40–50% of children required colectomy within 1–2 years [32,33]. Tacrolimus is not recommended as a long-term maintenance therapy in UC due to its long-term toxicity; however, it may be considered as a bridge therapy to TNF inhibitors, although some researchers express concerns regarding potential adverse effects when transitioning from a calcineurin inhibitor to a TNF inhibitor or vice versa [34,35].

Infliximab (IFX) is the leading agent used in ASUC for patients who do not respond to intravenous corticosteroids (IVCSs). Approximately 75% of children with ASUC treated with IFX as rescue therapy initially respond to the treatment. The introduction of IFX has comparably reduced the short-term colectomy rate from 40–70% to around 10–20% in children, and the colectomy rate after one year has decreased from approximately 60% to 18–22% [11].

Zitomersky et al. indicated that 20–40% of patients who initially responded to IFX treatment experienced side effects or the loss of response, often related to the formation of antibodies [36]. While TNF-alpha inhibitors generally have good tolerability, their efficacy may be limited by side effects such as infusion reactions. Additionally, serious side effects can occur [37]. The use of IFX in ASUC carries the risk of no response related to differing pharmacokinetics in this patient group. Patients with ASUC exhibit a faster clearance of IFX, likely due to elevated TNF levels, the increased activity of the reticuloendothelial system, and drug loss through leaky guts affected by heightened inflammation [38]. Additionally, significant protein loss in the intestines leads to hypoalbuminemia and the rapid clearance of infliximab through feces [39].

The major limitation of our study is its retrospective design and small sample size, which restricts the generalizability of the findings and emphasizes the need for future large-scale prospective studies.

## 5. Conclusions

Although this is a retrospective analysis, we believe that based on the above results, it can be hypothesized that pulse therapy may be one of the methods used for inducing remission in pediatric and adolescent patients with moderate to severe ulcerative colitis. The treatment’s response rate observed in our study is comparable to that achieved in studies with standard glucocorticoid dosing or cyclosporine [28,30]. However, pulse steroid therapy was associated with a shorter median treatment time and thus could show limited adverse effects compared to prolonged steroid therapy or cyclosporine treatment. In cases of no response to pulse therapy, it is possible to rapidly identify a group of patients requiring the initiation of second-line therapy, making high-dose methylprednisolone therapy a potential bypass for initiating subsequent therapeutic steps, such as the introduction of TNF-alpha inhibitors.

In the group analyzed in our study, the colectomy rate over the course of one year was 20%. However, among the patients who responded to treatment, none required colectomy during the year of observation. This may suggest that the use of high doses of methylprednisolone could be one of the methods for reducing the need for surgical intervention in patients with ASUC.

In low-income countries, where access to expensive biological therapies is limited, high-dose intravenous glucocorticoid treatment may represent an effective therapeutic option before considering surgical intervention for children with inflammatory bowel diseases. Such an approach could delay or even avoid the need for surgery while simultaneously controlling disease symptoms and improving patients’ quality of life [40].

A randomized prospective study should be planned to determine whether the median number of days to achieve a response to treatment, as demonstrated in our study, is less than that observed with standard glucocorticoid dosing and whether it facilitates a quicker decision to initiate second-line therapy. Shortening the induction time for remission is associated with a reduced hospital stay, lower treatment costs, and, most importantly, a decrease in discomfort and an improvement in the quality of life for patients.

## Figures and Tables

**Table 1:** Patient characteristics and short-term outcomes.

	Overall, *n* = 15	Non-Responders, *n* = 6	Responders, *n* = 9
Age	12.1 ± 3.6	11.3 ± 4.9	12.6 ± 2.7
Sex			
Female	8 (53%)	5 (83%)	3 (33%)
Male	7 (47%)	1 (17%)	6 (67%)
Disease severity			
PUCAI score at admission	71.3 ± 15.9	80.8 ± 4.9	65.0 ± 17.7
Moderate	3 (20%)	0 (0%)	3 (33%)
Severe	12 (80%)	6 (100%)	6 (67%)
Steroid-dependent ulcerative colitis	3 (20%)	0 (0%)	3 (33%)
Refractory ulcerative colitis	9 (60%)	6 (100%)	3 (33%)
Prior therapy			
Cyclosporin	1 (6.7%)	1 (17%)	0 (0%)
Thiopurine	2 (13%)	1 (17%)	1 (11%)
Budesonide	8 (53%)	3 (50%)	5 (56%)
5-ASA	13 (87%)	4 (67%)	9 (100%)
Disease duration			
First attack	3 (20%)	2 (33%)	1 (11%)
Exacerbation	12 (80%)	4 (67%)	8 (89%)
Months from diagnosis	4 (1–7)	1 (0–3)	4 (1–8)
Short-term outcomes			
PUCAI score after treatment	45.3 ± 26.3	75.0 ± 6.3	25.6 ± 9.2
Days to remission	4 (3–4)	NA	4 (3–4)
Days of therapy	4 (3–4)	3.5 (3–4)	4 (3–4)
PUCAI decrease	26.0 ± 20.7	5.8 ± 6.6	39.4 ± 14.7

Counts (%), medians (IQR), or means ± standard deviation are presented as appropriate for the data distribution.

## Data Availability

Data are available on request from the corresponding author. The data are not publicly available due to privacy or ethical restrictions.

## Abbreviations

The following abbreviations are used in this manuscript:

5-ASA	5-Aminosalicylic Acid
ADA	Adalimumab
ASUC	Acute Severe Ulcerative Colitis
CyA	Cyclosporine A
GCs	Glucocorticoids
IFX	Infliximab
IL	Interleukin
IQR	Interquartile Range
IVCSs	Intravenous Corticosteroids
PUCAI	Pediatric Ulcerative Colitis Activity Index
SD	Standard Deviation
TNF	Tumor Necrosis Factor
ESPGHAN	European Society for Paediatric Gastroenterology, Hepatology and Nutrition
